# Evidence for complementary learning systems within the hippocampus

**DOI:** 10.1098/rstb.2025.0243

**Published:** 2026-07-09

**Authors:** Dhairyya Singh, Anna C. Schapiro

**Affiliations:** Department of Psychology, University of Pennsylvania, Philadelphia, PA 19104, USA

**Keywords:** neuroscience, behaviour, cognition, neural network modelling, hippocampus, structure learning, generalization, prediction

## Abstract

Decades of research have established the hippocampus as central to episodic memory, but growing evidence suggests that it also contributes to structure learning, rapidly extracting regularities across experiences in support of prediction and generalization. These functions impose conflicting demands: episodic memory requires experiences to be separated, whereas structure learning requires integrating across them. The C-HORSE model, a biologically grounded neural network of the hippocampus, proposed an anatomical division of labour that resolves this tension. The trisynaptic pathway (TSP), characterized by high plasticity and strong pattern separation, supports rapid learning of individual episodes, consistent with prior theory and data. By contrast, the monosynaptic pathway (MSP), which learns more incrementally and with less separation, supports the extraction of regularities across experiences. This review evaluates how C-HORSE aligns with empirical findings on hippocampal involvement in structure learning. We review evidence from domains that benefit from quickly detecting regularities, including statistical learning, motor sequence learning, inference and category learning, surveying the role of the hippocampus in each and assessing evidence for functional dissociations across pathways in relation to model predictions. Together, this synthesis highlights strong convergence between C-HORSE and the empirical literature, identifies the MSP as a key contributor to structure learning and raises open questions for future experimental and modelling work.

This article is part of the theme issue ‘The role of hippocampal predictions in cognition: bridging perception and memory’.

## 1. Introduction

All learning systems must grapple with the stability–plasticity dilemma—a trade-off between durably storing knowledge for the long term and acquiring new information rapidly [1]. The Complementary Learning Ssystems (CLS) framework proposed an elegant solution: the brain resolves this dilemma by dividing learning across two systems, with the hippocampus using a pattern-separated code to quickly encode each new episode and the neocortex using an overlapping, distributed code to slowly extract the structure across many experiences, serving as our durable store of knowledge [2]. The CLS account has been highly influential and has received much empirical support since its proposal [3]. Yet, evidence from humans and other animals challenges the simple CLS dichotomy [4]. Much work has now shown that we are also able to learn structure—i.e. systematic relationships within or between events [2]—rapidly, often over the course of minutes [5–7]. The hippocampus, given its rapid and long-lasting plasticity [8], could be a candidate to support such learning, and indeed has been implicated in many forms of rapid structure learning [9–12]. But this raises a critical question: how can the hippocampus simultaneously support both episodic memory, which requires separating experiences to avoid interference, and structure learning, which requires integration across experiences?

To address this question, Schapiro *et al*. [13] proposed a biologically grounded neural network model of the hippocampus, C-HORSE (Complementary Hippocampal Operations for Representing Statistics and Episodes; figure 1), which advances the hypothesis that the hippocampus resolves these competing demands through a division of labour across its anatomical pathways. C-HORSE builds from prior hippocampal models that have been successful in explaining how the rapid learning and pattern-separated representations of the trisynaptic pathway [16,17] enable episodic memory [18–23]. These models provide a biologically detailed instantiation of the episodic memory-focused CLS account of the hippocampus [2] and do not propose a role for the hippocampus in the learning of regularities. C-HORSE inherits these prior models’ perspective on the TSP but additionally proposes a novel, complementary role for the monosynaptic pathway (MSP) in regularity learning. There is evidence that the MSP learns more incrementally [24] and develops more overlapping representations than the TSP [25], and C-HORSE proposes that these properties enable the rapid learning of statistics. C-HORSE thus proposes that a microcosm of the CLS-identified dichotomy manifests within the hippocampus itself, with the MSP serving as a neocortex-like system in complement to the TSP. On the dimensions of learning rate and representational overlap, the MSP is proposed to be situated between the TSP and neocortex, making it ideally suited to the rapid learning of across-episodic regularities on the timescale of minutes or hours—faster than the neocortical learning of regularities, unfolding across days, months and years but distinct from the one-shot episodic encoding capabilities of the TSP [26].

The two representational schemes in the MSP and TSP are subject to complementary trade-offs. Pattern-separated representations in the TSP store memories in largely non-overlapping sets of units. This enables high fidelity one-shot learning because new memories can be encoded immediately without danger of interfering with existing memories. However, since the units involved in each memory are distinct, these representations do not capture the regularities across experiences. Such regularities can in some cases still be uncovered, for example by iteratively retrieving individual memories based on content similarity to discover the relationships across them [27], but this is computationally expensive, especially as the set of memories in storage grows, and less time-efficient. Distributed representations in the MSP directly embed shared regularities; i.e. experiences that share features recruit overlapping units, and therefore regularities are cached during learning (we use the term *distributed representation* in the sense defined by [28], in which each unit responds to many entities). This overlap supports efficient prediction and generalization but makes the system vulnerable to interference [29], requiring slower, interleaved learning to adjust representations until all memories can be accommodated.

C-HORSE incorporates key aspects of the biological circuitry of the hippocampal TSP and MSP (figure 1). The TSP in the model consists of superficial entorhinal cortical (EC_in_) projections to subfield dentate gyrus (DG) and cornu ammonis 3 (CA3), a DG projection to CA3, recurrent CA3 connections, and finally a CA3 projection to CA1. The MSP includes a direct projection from EC_in_ to CA1 and from CA1 to deep EC (EC_out_). EC_out_ projects back into EC_in_ in the model, enabling ‘big loop’ recurrence in the hippocampal system; i.e. the feeding of retrieved information back into the system [27]. More recently, a version of the model was extended from Schapiro *et al*. [13] to incorporate empirically observed differences in the topographic targeting of projections from CA3 and superficial EC to CA1 along its proximodistal axis [30–32]. As a simplified implementation of this differential topography, CA1 was split into two parts (proximal/distal) such that the TSP ran through proximal CA1 (pCA1) and the MSP through distal CA1 (dCA1) [33,34]. This separation reduces interference between pathways, allowing better isolation of the contributions of each pathway.

C-HORSE has been able to account for hippocampal involvement in a range of rapid novel structure learning problems, including statistical learning, associative inference and category learning, by exploiting the incrementally learnt distributed representations of the MSP [13,14,33–35]. A central prediction of our account is that the hippocampus should be engaged across these and other paradigms that benefit from the rapid extraction of regularities across experiences—a departure from traditional views of the hippocampus as a system dedicated to episodic memory through preservation of experiences as discrete snapshots. In §2, we introduce the architectural and computational attributes that form the foundation of C-HORSE. We describe how the model instantiates key properties of the hippocampal pathways—the sparse, high-learning-rate architecture of the trisynaptic pathway and the dense, lower-learning-rate architecture of the monosynaptic pathway—and how these features jointly enable separation and regularity extraction. In §3, we turn to examining the involvement of the hippocampus in a range of paradigms that benefit from the rapid extraction of regularities and assess the extent to which known functional dissociations in the contribution of hippocampal subcircuits to structure learning align with the predictions of C-HORSE.

## 2. Architecture and computational mechanisms

The hippocampal TSP is known to have one-shot learning capabilities and to develop sparse representations suited for performing pattern separation—the segregation of similar inputs into distinct representations, as well as recurrent lateral connectivity in CA3 that supports pattern completion—the reconstruction of stored representations from partial or degraded cues [17,21,24,36]. A range of convergent findings across rodent electrophysiology and human neuroimaging experiments have implicated the biological pathway EC → DG in pattern separation [16,37–47]. This pattern separation is driven by the vast expansion in the number of DG cells in comparison with EC input cells [21,48] and high levels of inhibition within DG [49,50]. The highly sparsified output from DG, with only 1–2% of units firing at a time [51], is projected onto CA3 (DG → CA3), which rapidly forms memory attractors (stable self-reinforcing population activity patterns corresponding to individual experiences) in its recurrent circuitry (CA3 → CA3; [52–55]), as quickly as in one shot [24,56]. Subsequently, CA3 performs pattern completion given cues that are similar to previously encoded inputs by reconstructing these attractors [57,58].

C-HORSE, in line with its predecessors [19,21–23,59] as well as other related models [18], implements analogous pattern separation and completion in the simulated TSP circuitry. The number of units in the C-HORSE DG is vastly greater than the number of EC units, mimicking the expansion in the biological circuit. EC → DG, EC → CA3, as well as DG → CA3 have highly sparse connectivity, with input units probabilistically targeting only a fraction of units in each layer. Both DG and CA3 also have high levels of inhibition. These properties serve to orthogonalize inputs from EC. EC → DG, EC → CA3, DG → CA3 and CA3 → CA3 have high learning rates, enabling rapid encoding of inputs. The recurrent connections in CA3 represent inputs as attractors in its auto-associative network and enable pattern completion during retrieval.

The hippocampal MSP has contrasting computational properties to the TSP. Unlike subfields DG and CA3, CA1 representations show baseline overlap even for dissimilar inputs, with this overlap scaling linearly with input similarity across humans and rodents ([17,25,43,60–63]; cf. [64]). In other words, while DG and CA3 make even similar inputs distinct, CA1 maintains a representational continuum that preserves the similarity structure present in EC inputs. Activity in CA1 has also been shown to be less sparse in comparison with CA3, with one study estimating that CA1 has approximately twice the activity level (35% versus 18% [63]). Finally, the MSP, in contrast to the TSP, performs more incremental learning [24,65], and CA1 activity remains stable for longer as the environment changes compared with CA3 activity ([52]; cf. [66]).

The C-HORSE MSP, in accordance with the properties of the biological subcircuit, incorporates dense connectivity, lower learning rate and inhibition, allowing it to learn more incrementally and develop distributed representations. The lineage of models from which C-HORSE developed [22,23,59] considered the role of the CA1 to be a mere ‘translator’ between the sparse representations of the TSP and overlapping representations of EC, and therefore did not allow MSP to learn during task training (although other models outside of this lineage have trained MSP connections; e.g. [18,67]). Instead, models in this lineage typically pre-trained EC ↔ CA1 weights to instantiate a stable mapping between EC input and EC output representations, and then froze the weights during memory simulations, thereby imposing a fixed cortical interface to which trisynaptic representations were required to align during learning. However, empirical findings have demonstrated independent learning in the EC ↔ CA1 pathway that can support behaviour even in the absence of input from CA3 [24,68]. How does CA1 incorporate input from both CA3 and EC, and allow each pathway to learn without interference from the other? Electrophysiological work has found that activity in CA1 is more strongly driven by EC or CA3 across separate phases of the 3–8 Hz theta rhythm [69,70]. Ketz *et al*. [19] incorporated this segregation into a hippocampal model in this lineage for the first time, enabling independent learning in both MSP and TSP despite their shared CA1 target. C-HORSE adopts this theta-based learning scheme. There is also evidence of a topographic gradient in the organization of projections from CA3 and EC along the proximodistal axis of CA1 [31,32], which was recently incorporated into a variant of C-HORSE [33,34]. This allows inputs from EC and CA3 to be separated into different parts of CA1 (CA3 → pCA1, EC → dCA1), further enabling independent learning as well as independent expression of each pathway’s representations. There is additional evidence for anatomical segregation of the two pathways along the radial axis of CA1, where EC and CA3 appear to differentially influence deep and superficial CA1, respectively [71,72].

The hippocampal MSP also has intrinsic properties related to representing the temporal organization of experience. The EC → CA1 pathway has been implicated in temporal association memory in rodents ([73–75]; cf. [76]). CA1 neurons in both rodents and humans have been identified as ‘time cells’ that fire sequentially during experiences [77–79], with related temporal coding observed in EC [80,81]. A related phenomenon has been observed in which the rodent CA1, but not CA3, is implicated in representing temporal intervals across hours and days via gradually drifting autocorrelated population activity [82–84]. In humans, CA1 has been shown also to represent temporal intervals [85] and temporal context [86]. It has been argued that both sequential firing in the short term and drifting autocorrelation over longer periods in CA1 might be generated by EC as a function of evolving experience, as opposed to an effect of time *per se* [87,88]. Under this account, CA1 ‘temporal representations’ are built primarily based on structured inputs from EC rather than being self-generated. Consistent with this account of EC → CA1 function, the C-HORSE CA1 relies on input correlations from EC to build its distributed representations. While EC is not actively trained in current versions of C-HORSE—i.e. it is a non-learning input/output layer—we assume that EC representations are sensitive to similarity structure in sensory inputs [89,90] and drive CA1 representations.

## 3. Hippocampal involvement and subregion dissociations in rapid structure learning

Evidence across a range of paradigms has shown that humans can rapidly learn structure, enabling prediction and generalization. Here we review the extent to which the hippocampus contributes to learning across these paradigms. We also survey work in both animals and humans that has examined subregion dissociations in contributions to structure learning and evaluate alignment with the C-HORSE account.

### (a) Statistical and motor sequence learning

#### (i) Evidence of hippocampal involvement

Although the term *statistical learning* can be used to describe the process of extracting any kind of statistic across experiences, the experimental literature typically uses the term in reference to a specific set of paradigms in which participants are incidentally exposed to continuous streams of stimuli with hidden temporal or spatial co-occurrences, usually pairs or triplets of stimuli that tend to occur together. Behaviourally, humans often show evidence of sensitivity to these regularities after only minutes of exposure [91–95]. Anatomical studies in children and adults have found that hippocampal volume relates to statistical learning performance [96,97]. Functional neuroimaging studies of temporal visual statistical learning have found that activity in the hippocampus tracks structured versus random sequences [98,99] and relates to the anticipation of predictable objects [100,101]. Studies of statistical learning of syllable sequences have implicated the superior temporal gyrus and inferior frontal gyrus but not the hippocampus [102–105], indicating potential differences across modalities, although paradigms using sequences of tones have also implicated the hippocampus [106–108]. More work will be needed to understand whether differences in stimuli or potentially in imaging methodology (acquisition and/or analysis) might explain the different findings across these studies [109]. Representational similarity analyses, which examine correlations in distributed patterns of neural activity, have also shown that object representations become more similar in response to reliable regularities in both auditory and visual domains [11,110–113]. When the environment contains reliable regularities, the hippocampus appears to reshape its representational space so that related item representations become integrated.

Studies of patients with damage to the hippocampus or the medial temporal lobe (MTL)—a region that includes the hippocampus as well as surrounding cortices—can provide more direct causal evidence for its role in statistical learning. Two studies comparing amnesic patients with healthy controls across a multi-modal battery of statistical learning tasks (shapes, syllables, scenes, tones) and both pair and triplet temporal regularities, found deficits in patient performance compared with controls, with patients performing at or near chance levels [114,115]. The deficits were especially clear in the case of pair learning, as controls were robustly above chance in this case. A third study using visual triplets found that while patient performance was at chance, control participants also frequently performed at chance, making it more difficult to draw strong conclusions [116]. A patient study using the Artificial Grammar Learning paradigm suggested that sensitivity to chunk strength —the familiarity of co-occurring fragments—is impaired with hippocampal damage, consistent with its role in statistical learning [117]. These studies used post-learning assessments of familiarity with regularities, however, which leaves open the possibility that the patients do learn regularities in some form during initial exposure but are unable to retain them through the assessment. Future patient work will be needed using online measures of learning such as reaction time or neural entrainment to evaluate this possibility [118].

The hippocampus also contributes to spatial statistical learning, as demonstrated in the contextual cueing paradigm [119]. Contextual cueing involves finding a target shape in a display, where the target’s location is predictably tied to the surrounding distractor arrangement. The hippocampus has been consistently implicated in neuroimaging studies of the contextual cueing paradigm, with hippocampal activity shown to distinguish between random and structured arrays [94,120–123]. Patients with MTL damage have also been found to be impaired at the task [124].

While statistical learning has typically been studied with passive perceptual tasks, there is also a parallel sequence learning literature investigating the implicit learning of motor sequences. The most common paradigm—the serial reaction time task (SRTT)—requires participants to make speeded responses to stimuli appearing in fixed locations, with the location varying systematically across trials [125]. The SRTT has been found to engage many brain areas, including parietal, motor cortical, prefrontal, cerebellar, striatal and medial temporal regions [126–136]. There is debate as to whether hippocampal integrity is necessary for performing this task, with some accounts arguing that it does not contribute to the learning of simple ‘first-order’ sequences, i.e. sequences in which individual trials predict the next, but contributes to the learning of more complex ‘second-’ or ‘higher-order’ sequences requiring the tracking of multiple recent transitions [131,137]. Consistent with this distinction, both human amnesic patients and rats with hippocampal lesions show deficits on second-order but not first-order SRTT [137,138], and fMRI work in healthy adults shows robust hippocampal engagement during the learning of higher-order structure in the SRTT [133]. While there is impairment in amnesic patients compared with controls, performance for the patients is still above chance [137], which is in contrast to patient performance in statistical learning, which tends to be at or near chance [114,115]. Thus, while the hippocampus contributes to the learning of complex motor sequences, it is not the only region supporting this learning, with motor-oriented regions like striatum and motor cortex probably playing a central role [139,140], and it does not seem as critical for simple motor sequences.

#### (ii) Pathway dissociations

The C-HORSE MSP, but not TSP, is highly sensitive to the regularities in statistical learning paradigms [13,35]. The overlapping representations and relatively slower learning rate of the MSP are ideally suited to integrating co-occurrence statistics across experiences on this timescale. These properties are common to many neural network modelling approaches that have successfully accounted for the learning of statistical structure [141–149]. The key hypothesis put forward by C-HORSE is that the hippocampus, and in particular the MSP, should be critical for this learning.

Wang and colleagues found that a patient with specific damage to the DG was successful in implicit statistical learning [47], which, in combination with the amnesic findings above [114,115], suggests that hippocampal contributions to statistical learning are supported by other subfields. In another study, this group found that healthy ageing is associated with a decline in pattern separation, assumed to be related to weakening DG function, but not in implicit statistical learning, supporting the anatomical dissociation between these functions [150]. Schapiro *et al*. [11] observed that region CA1 was sensitive to probabilistic sequences generated from a graph with community structure, although prior work with simpler pairwise transition structure showed broader recruitment across hippocampal subregions [111]. One study in mice found that CA1 but not DG or CA3 contributes to the incremental updating of learnt probabilities in a probability reversal learning task ([151]; figure 2A). While existing evidence is thus broadly consistent with C-HORSE, more empirical work will be needed to evaluate specific pathway contributions to statistical learning.

Beyond the probabilistic paradigms of statistical learning, there is also a literature exploring fixed, demarcated sequences. The rodent CA1 has been found to critically support temporal sequence memory in such paradigms [156,157]. Schapiro *et al*. [13] noted that the computational mechanisms employed by the MSP and TSP suggest differing capabilities in sequential learning. The TSP is predicted to be better-suited to learning sequential information that is fixed across presentations (consistent with [158]) or only presented once, while the MSP would be required for sequences that are probabilistic and require extraction of transition structure across presentations. If the fixed sequences are repeated, however, the MSP is also predicted to learn them incrementally, and would show sensitivity to the sequential structure and support behaviour. This is consistent with CA1 being implicated in sequence learning quite broadly in the literature [159]. One recent study in mice found that implicit behavioural signatures of sequence learning were critically impaired following inactivation of the dorsal CA1 [108]. This study used fixed, demarcated sequences, but learning was incidental and required sensitivity to event frequency across exposures, thus having elements of both kinds of sequential learning. The learned representations appeared to be abstracted from the specific sensory features of the trained sequences, potentially supporting generalization to novel stimuli and consistent with learning in the C-HORSE MSP.

### (b) Inference

#### (i) Evidence of hippocampal involvement

Inference requires finding connections across discrete events, for example recognizing that if A and C are each associated with B (AB, BC) then they are indirectly related (AC; associative inference); if A is preferable to B (A > B) and B is preferable to C on the same dimension (B > C), then A must also be preferable to C (A > C; transitive inference); and if A and B lead to some outcome X (A/B → X) and A is shown to lead to outcome Y (A → Y), B might also lead to Y (B → Y; acquired equivalence). Hippocampal lesions in rodents produce critical impairments in both associative inference [160] and transitive inference [161]. Likewise in humans, hippocampal and broader MTL damage or atrophy have been linked to critical impairments in associative inference [162] and acquired equivalence [163]. Across tasks and species, premise memory (AB) is typically spared while inference or transfer is specifically impaired.

#### (ii) Pathway dissociations

Two broad accounts have been proposed for hippocampal contributions to inference, with a particular focus on mechanisms supporting associative inference [7,34,164]. Retrieval-based accounts posit that episodes are encoded separately and linked only at retrieval through an active process of recombining related memories [27,165,166], whereas encoding-based accounts propose that related episodes are integrated as they are learnt, so that inference follows automatically at retrieval [34,90,167–170].

The retrieval-based perspective has been formalized in the Recurrency and Episodic Memory Results in Generalization (REMERGE) model [27], which proposes that the hippocampus encodes pattern-separated representations of discrete episodes and iteratively links episodes through ‘big loop’ recurrence to make inference judgements. In C-HORSE, the TSP, in concert with EC_out_ → EC_in_ connections, can also instantiate this strategy. Two theoretical perspectives have been proposed to explain how the hippocampus might execute an encoding-based strategy: one is the proposal that the distributed representations of the MSP allow it to incrementally develop integrated representations that enable the ‘caching’ of commonalities at encoding [34]. Another is the integrative encoding account, which posits that the encoding of a new episode drives the similarity-based retrieval of episodes with overlapping mnemonic content, which can then be integrated with the new episode [7,170]. The integrated representations that both accounts propose then allow for inference without additional processing at retrieval. While both encoding-based accounts predict integrated representations, integrative encoding relies on the pattern completion-driven retrieval in the TSP, which can support one-shot integration or integration under conditions of blocked presentation (because the TSP can avoid the interference that would otherwise be induced by blocking), whereas the C-HORSE instantiation of this strategy relies only on the distributed code of the MSP, and it requires more exposures presented in interleaved order.

Several neuroimaging studies have investigated these strategies, with some cited as providing support for an encoding-based strategy [170–172] and others for a retrieval-based strategy [166,173], but a clean distinction is tricky because the spreading activation proposed by REMERGE can closely mimic integration in the fMRI BOLD signal [27]. One way to distinguish the MSP-based strategy from both integrative encoding and REMERGE is to take advantage of the different sensitivity to presentation order, as only the MSP requires interleaved learning. A series of behavioural experiments using this approach found signatures of integration that emerged only after interleaved learning, which provides evidence for use of this strategy [34].

All three of these strategies are probably employed by the hippocampus under different conditions, and disambiguating when each is engaged will be a critical goal for future work. Time-resolved methods such as electroencephalography (EEG), intracranial electroencephalography (iEEG), and magnetoencephalography (MEG), coupled with representational analysis tools, may be especially useful in determining whether integration signatures emerge as static representational changes or transient iterative recombinations. To our knowledge, no computational model currently instantiates all three strategies. C-HORSE instantiates a REMERGE-like strategy via computations in the TSP and big loop recurrence, as well as MSP-based integration [13], but it would be fruitful to additionally allow it to support integrative encoding by enabling encoding updates driven by reinstated content. Such a unified model may be helpful in identifying the specific conditions under which each strategy is used.

### (c) Category learning

#### (i) Evidence of hippocampal involvement

Category learning involves the extraction of rules or features on the basis of which entities can be grouped. Humans can rapidly learn new categories in a sample-efficient manner and generalize category structure to novel situations effortlessly [164,174,175]. While many brain areas, including the basal ganglia, parietal regions and prefrontal regions, are known to be involved in category learning [176,177], neuropsychological evidence from amnesiacs and lesion studies in rodents have also implicated the hippocampus [178–181]. Neuroimaging studies investigating a wide range of category learning paradigms have also indicated hippocampal involvement in rapid category learning and generalization [174,175,182–185] and single cells in the monkey hippocampus become tuned to novel categories after learning [186]. Overall, there is strong evidence that the hippocampus makes some contribution to category learning.

#### (ii) Pathway dissociations

Sučević & Schapiro [14] applied C-HORSE to several category learning paradigms to provide an account of how the pathways of the hippocampus may contribute. The paradigms involved learning the visual features of distinct family-resemblance categories [187], learning probabilistic categories with uncertainty in how well individual features predict category membership (weather prediction task [188]) and learning categories with across-category feature sharing and classification depending on an arbitrary learnt boundary [175]. Across tasks, the MSP was critical for category learning and generalization, employing its distributed representations and incremental learning to extract feature regularities over trials. The TSP, by contrast, formed separated representations of exemplars and was crucial for memory for atypical or idiosyncratic features. An fMRI study using the family-resemblance task found that CA1 (but not CA3/DG) indeed represented the category structure, as found in the model [189]. Another study found a correlation between TSP white matter integrity and exception learning, consistent with C-HORSE’s account of TSP contributions [190]. Recent work employed a novel joint Diffusion Weighted Imaging (DWI) and fMRI approach to identify voxels within CA1 that are preferentially driven by EC or CA3 axonal projections and found that the EC-driven (i.e. MSP-targeted) voxels tracked the learning of shared category features, whereas CA3-driven (i.e. TSP-targeted) voxels tracked the learning of exceptions [30]. While more empirical evidence is needed, especially in non-human animals, the existing evidence is strongly consistent with the pathway dissociations in category learning proposed by C-HORSE.

### (d) Contextual fear conditioning and spatial learning

Several studies have examined relevant functional dissociations within the hippocampus in contextual fear conditioning and other spatial learning paradigms. Nakashiba *et al*. [24] blocked the CA3 → CA1 projection in mice, thereby knocking out TSP input to CA1, and tested the animals on two hippocampus-dependent tasks (figure 2B). The first was a one-shot contextual fear conditioning task in which mice learnt to associate an environment with a footshock. Upon re-exposure to the same environment, the TSP-silenced mice showed impaired re-expression of the fear memory, indicating an impairment in associative learning (figure 2B(i)). The second task was an incrementally learnt Morris Water Maze spatial learning task in which mice were placed in a pool of water and learnt to swim to a hidden platform to escape the water. The location of the platform was held constant and, over the course of several days, the TSP-silenced mice improved their performance at the same rate as controls (figure 2B(ii)). Another study found a rapid learning impairment after blocking CA3 plasticity in a one-shot variant of the Morris Water Maze, ruling out task-type confounds [56]. This dissociation indicates that the CA3 → CA1 projection is not necessary for incremental learning but is required for one-shot learning, supporting dissociations in learning rates across the two pathways of C-HORSE.

Two recent contextual fear conditioning studies in mice used optogenetic silencing of hippocampal circuit elements to directly probe MSP and TSP contributions to memory specificity. Atucha *et al*. [152] demonstrated that blocking CA3 during retrieval caused context-specific freezing behaviour to become non-specific, with mice also freezing in another similar context, implicating the CA3 in maintaining precise detailed memory. Blocking CA1 abolished fear expression completely, consistent with its role in conveying all hippocampal output to EC (figure 2C). McKenzie *et al*. [153] found that silencing the CA3 → CA1 projection but not medial EC → CA1 during learning shifted CA1 towards lower-dimensional memory representations and produced a parallel loss of specificity in freezing behaviour. These findings are highly consistent with the C-HORSE hypothesized division of labour—they demonstrate causally that the TSP but not MSP is necessary for high-resolution detailed memory and suggest that the MSP supports lower-resolution, gist-like memory (figure 2D).

### (e) Dissociations along the longitudinal axis

A large literature has identified functional differences along the hippocampal longitudinal axis in both humans and other animals. In rodents, this axis runs ventrally to dorsally and in humans and monkeys anteriorly to posteriorly [191–194]. In humans, the anterior hippocampus contains proportionally more CA1–3, whereas the posterior hippocampus contains proportionally more DG [195], raising the possibility that longitudinal axis differences can serve as a coarse proxy for subfield differences. The precise distribution of CA1 and CA3 volume across anterior and posterior segments is unclear owing to variance in segmentation methodologies, which makes it difficult to cleanly associate longitudinal differences to subfield-specific functions. The anterior-most tip of the hippocampus, however, consists almost entirely of CA1 and subiculum, leading some to argue that neuroimaging studies should segment this region separately to better constrain functional interpretations [196,197].

This caveat notwithstanding, anterior hippocampus has been consistently implicated in statistical and sequence learning [11,98,101,198]. A neuroanatomical study with children and adults found that anterior hippocampus volume correlated significantly with measures of statistical learning, although an exploratory within-head subfield analysis suggested that this effect was driven by CA2/3 and subiculum rather than CA1 [97]. Anterior hippocampus has been implicated in forming gist-like or prototype-based representations and integrating across related experiences, while posterior hippocampus has been linked to detailed episodic memory and fine discrimination between experiences [6,9,12,175,182,183,192,199–203]. These distinctions are broadly consistent with the C-HORSE account, but more work is needed to determine precisely how longitudinal-axis findings map to subfield-specific contributions.

### (f) Dissociations over development

Infants are able to perform a variety of structure-learning tasks, including statistical learning [91,92,98,204], category learning [205,206] and some forms of inference [207–209], despite well-documented limitations in episodic memory and hippocampal function [210]. The hippocampus is not fully developed in early childhood, and in fact goes through a protracted developmental trajectory stretching into adolescence, with corresponding slow maturation of episodic memory capabilities [211–218]. To the extent that these structure-learning tasks are hippocampally dependent, the slow developmental trajectory raises the question of how young children, especially infants, can be so adept at them. C-HORSE’s proposed functional dissociation across hippocampal pathways offers a resolution to this puzzle, as there is evidence that the two pathways mature at different rates. CA1 is the first subfield to mature in childhood—by the age of two years, while DG and CA3 continue maturing into adolescence [210]. An anatomical analysis of the monkey CA1 also found that the laminar region, which receives projections from EC (EC → CA1; stratum lacunosum-moleculare), matures before the region that receives projections from CA3 (CA3 → CA1; stratum radiatum), indicating a faster postnatal development of the MSP compared with the TSP [219]. These divergences in maturation rates could explain why infants are able to perform the proposed MSP-supported tasks but not TSP-supported tasks.

### (g) Representational change

Human neuroimaging studies using representational similarity analyses have provided important insights into how hippocampal subfields form and modify representations, bearing on the proposed representational dissociations between the MSP and TSP. Consistent with the C-HORSE account, CA1 but not DG/CA3 shows evidence of integrating representations of: objects belonging to the same category [189], indirectly related items in associative inference ([154]; figure 2E), objects encountered in the same spatio-temporal context ([155]; figure 2F), as well as objects belonging to the same temporal community [11].

By contrast, DG/CA3 subfields separate representations of: indirectly related items in associative inference [154], objects in the same spatiotemporal contexts [155] and highly similar objects in a memory association paradigm that induces competition between them [220]. While C-HORSE proposes orthogonalization in DG/CA3, some studies report the phenomenon of repulsion, where differentiation drives representations below baseline orthogonalization levels [221,222]—a phenomenon that C-HORSE does not currently exhibit. Intriguingly, two studies have shown that statistical learning induces integration for objects with strong statistical relationships or high visual similarity and repulsion for objects with weak statistical relationships or low visual similarity, but these effects are consistent across hippocampal subfields as well as broader MTL areas [111,113]. Extensions to C-HORSE will be needed to explain these repulsion effects, perhaps integrating non-monotonic plasticity mechanisms [223,224].

## 4. Other frameworks and open questions

Structure is ubiquitous in our daily life, and we are adept at rapidly exploiting it to make better predictions and generalize effectively to novel situations. Schapiro *et al*. [13] proposed that the hippocampus plays a crucial role in this behaviour, with a special role for the MSP. In reviewing the literature on hippocampal structure learning, we found strong consonance with the C-HORSE proposal, though we also identified many areas in need of further study as well as specific empirical phenomena that current instantiations of C-HORSE do not accommodate.

Several other frameworks have proposed distinct functional roles for the MSP and TSP. One influential account, advanced by Hasselmo and colleagues [70], emphasizes theta-phase segregation as a mechanism for alternating encoding and retrieval modes, with EC → CA1 input dominating during the theta trough to drive encoding of new sensory information and CA3 → CA1 input dominating during the theta peak to support retrieval through pattern completion. Under this view, the MSP largely provides sensory input for episodic encoding rather than learning separate representations in its own right. Related ‘comparator’ models situate CA1 as a site that detects mismatches between EC input and retrieved CA3 patterns, signalling novelty or prediction error [18,225–227]. Another set of models—including the predecessors of C-HORSE—conceptualize the role of CA1 as a decoder or intermediary, merely translating between the pattern-separated representations of the TSP and the overlapping representations of EC [19,22,23,59]. Finally, a recent line of work on behavioural timescale plasticity in CA1 [228,229] proposes that EC-driven plateau potentials integrate temporally extended experiences and modulate CA3-driven plasticity, casting the MSP as a modulatory system that shapes TSP-based episodic learning. While these accounts are not necessarily mutually incompatible with one another or with C-HORSE, compared with these accounts, C-HORSE attributes a more computationally autonomous role to the MSP: rather than providing a supervisory, modulatory, or translatory signal, it actively learns distributed representations that capture regularities across experiences and can independently support generalization.

Future work leveraging more fine-grained methods will be essential for distinguishing among these accounts. Projection-specific, rather than region-wide manipulations, via optogenetic or chemogenetic tools (e.g. [153]) will be important to identify the independent learning contributions of each pathway, and calcium imaging or single cell recordings could be employed to reveal the representational structure underlying learning. Recent advances in DWI [230,231], especially in combination with fMRI [30], offer the potential to link anatomical connectivity with representational organization and behaviour, enabling pathway-level tests of these different functional proposals in humans.

Our account of hippocampal structure learning is consistent with accounts that conceptualize the hippocampus as building a cognitive map, representing relationships among experiences across spatial, temporal and conceptual domains [232–235]. Within this relational learning landscape, the MSP provides a useful strategy in contexts where its overlapping, distributed code confers a particular advantage—for example, statistical learning paradigms that demand rapid integration across related experiences to form predictive representations. Additionally, we do not claim that the MSP is the sole substrate of structure learning. Other hippocampal circuits (TSP) and extra-hippocampal systems (e.g. striatum) also contribute via distinct mechanisms and may be better suited in particular settings. Our account, therefore, specifies the conditions under which MSP computations play a privileged role in rapid structure learning, while situating them within a broader repertoire of strategies for parsing and representing experience.

Besides the questions we have already identified across the previous sections, two broader areas stand out as critical directions for future work. First, while C-HORSE proposes that the hippocampus builds contrasting representations along its pathways, it remains unclear how and under what conditions these distinct representations influence behaviour. One possible candidate for dynamic control over this influence is the interaction between the hippocampus and medial prefrontal cortex [236,237], which has bidirectional anatomical connectivity with CA1—where the pathways converge—and has been shown to modulate the representational structure of CA1 in a task-adaptive manner [238–241]. It will be crucial for future modelling and empirical work to explore this interaction as well as other possible candidates in order to evaluate whether and how the brain can deploy the best representation for a given task. Second, a fuller account of hippocampal computation will require the development of a more detailed architecture incorporating subfields such as CA2 and subiculum, as well as accounting for the heterogeneous connectivity profiles of individual hippocampal subfields to other parts of the medial temporal lobe and external structures [196,230,242,243].

In conclusion, converging empirical findings lend strong support to the core principles of C-HORSE, yet much work remains to delineate the functions of the pathways of the hippocampus. We hope the questions identified in this review will inspire further empirical and modelling efforts.

## Figures and Tables

**Figure 1.**
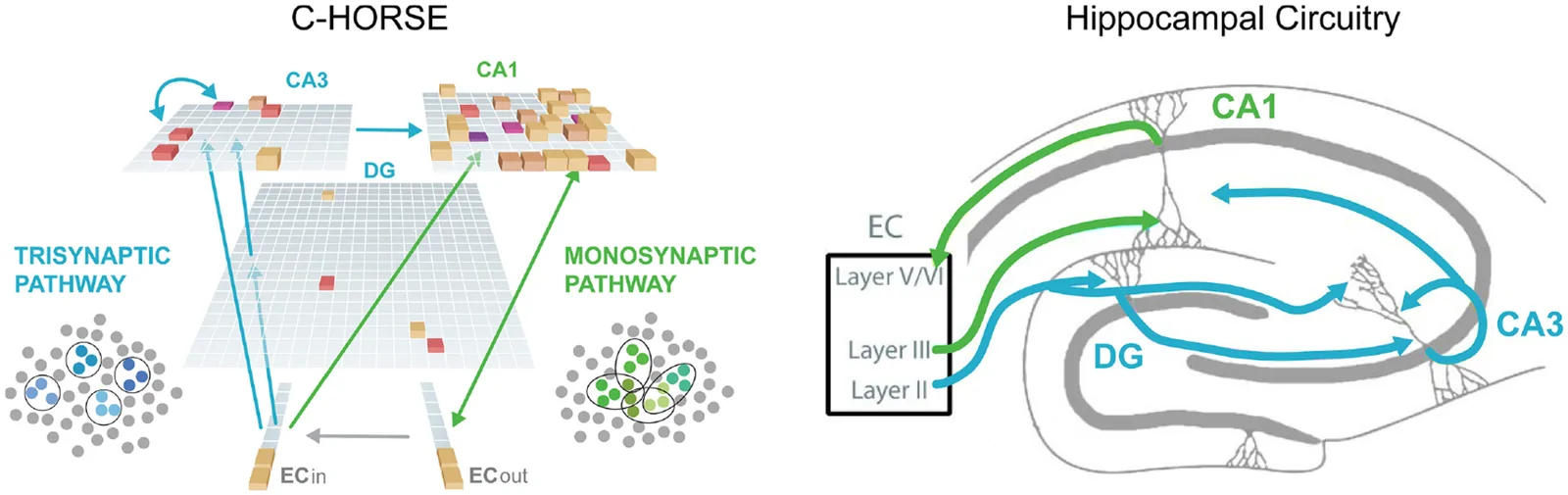
C-HORSE architecture. C-HORSE implements the two-pathway circuitry of the hippocampus. It proposes complementary learning functions across these pathways, with the trisynaptic pathway rapidly forming pattern-separated representations (separate blue unit pools) and the monosynaptic pathway more incrementally forming overlapping representations (overlapping green pools). CA1, cornu ammonis 1; CA3, cornu ammonis 3; DG, dentate gyrus; EC_in_, superficial entorhinal cortex; EC_out_, deep entorhinal cortex. Figure adapted from [14] and [15].

**Figure 2.**
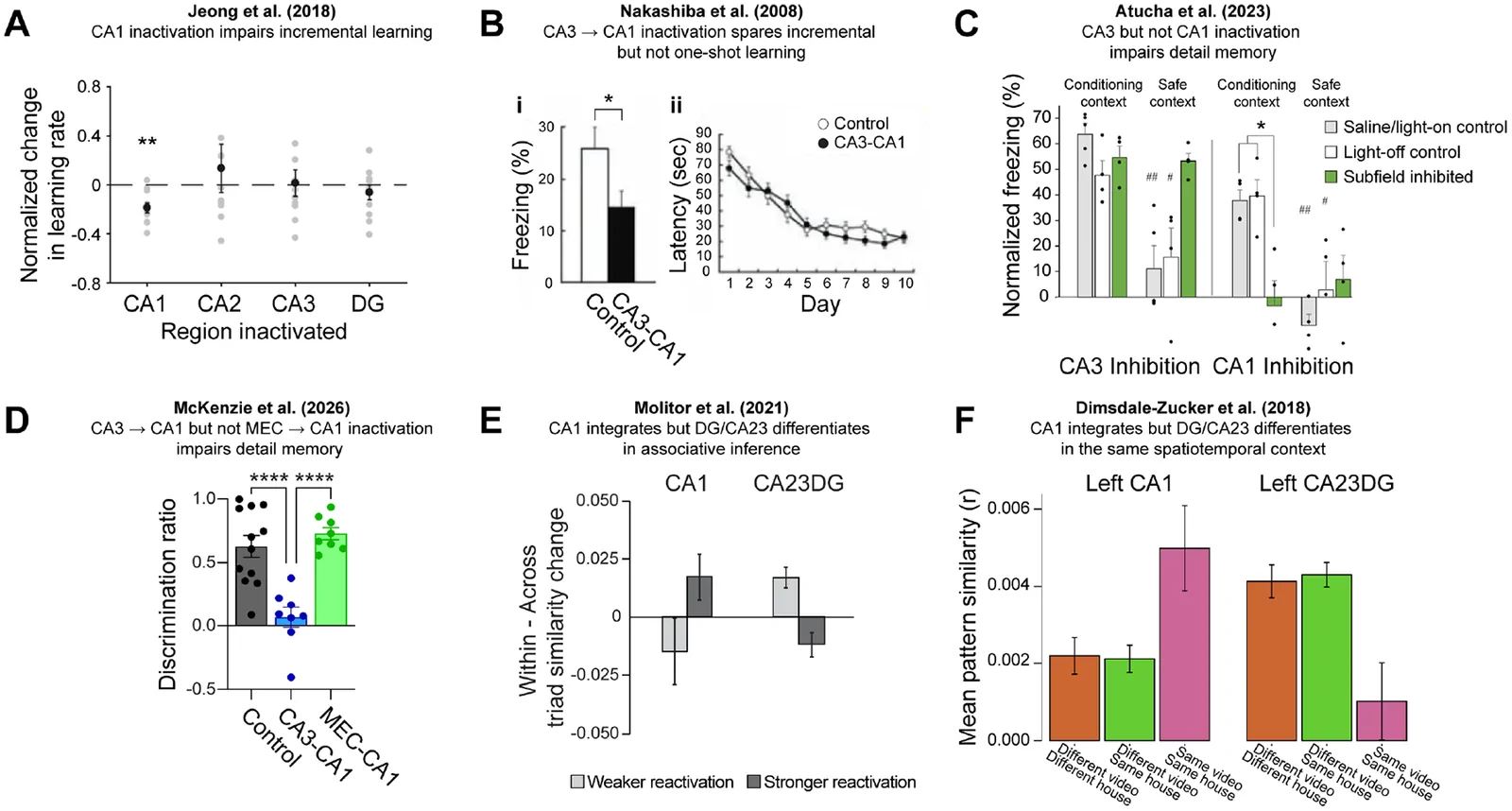
Human and rodent findings on distinct pathway functions. (A) Jeong *et al*. [151] trained mice to perform probability reversal learning, which required integrating recent reward outcomes in order to make decisions to maximize future reward. They found that CA1 but not CA2, CA3 or DG inactivation led to a significant slowdown in the ability to learn incrementally from recent outcomes. Adapted under the CC BY 4.0 license. (B) Nakashiba *et al*. [24] inactivated the CA3 → CA1 pathway in mice and observed impaired one-shot learning (i) but intact incremental learning (ii). Adapted with permission from AAAS. (C) Atucha *et al*. [152] inactivated CA3 or CA1 during retrieval after context fear conditioning and found that only inactivating CA3 made the fear memory nonspecific, while inactivating CA1 abolished the memory. Adapted under the CC BY 4.0 license. (D) McKenzie *et al*. [153] inactivated hippocampal projections during learning in a contextual fear conditioning task and found that inactivating CA3 → CA1 but not MEC → CA1 caused a loss of memory specificity. Adapted with permission. (E) In Molitor *et al*. [154], human participants performed an associative inference task that involved encoding premise AB and BC pairs and later inferring AC relationships. They found differentiation in DG/CA23 and integration in CA1 for the same indirectly related AC items when the A item was strongly retrieved at the point of BC encoding. Adapted under the CC BY-NC-SA 3.0 license. (F) In Dimsdale-Zucker *et al*. [155], participants watched videos of objects in different contexts (houses) and found differentiation in DG/CA23 but integration in CA1 for objects from the same video and context. Adapted under the CC BY 4.0 license.

## Data Availability

This article has no additional data.
